# “I Want to Do Something” – Exploring What Makes Activities Meaningful for Community-Dwelling People Living With Dementia: A Focused Ethnographic Study

**DOI:** 10.1177/10497323241239487

**Published:** 2024-04-22

**Authors:** Emma Harding, Mary Pat Sullivan, Paul M. Camic, Keir X. X. Yong, Joshua Stott, Sebastian J. Crutch

**Affiliations:** 1 Dementia Research Centre, Department of Neurodegenerative Disease, UCL Queen Square Institute of Neurology, 4919 UCL, London, UK; 2 School of Social Work, Faculty of Education and Professional Studies, 6057 Nipissing University, Nipissing, ON, Canada; 3 UCL Division of Psychology and Language Sciences, Department of Clinical, Educational, and Health Psychology, 4919 UCL, London, UK

**Keywords:** ethnography, dementia, meaningful activities, neurocognitive disorders, qualitative research

## Abstract

Supporting ageing in place, quality of life, and activity engagement are public health priorities for people with dementia. The importance of maintaining opportunities for meaningful activities has been widely acknowledged for those with dementia in long-term care, but little is known about what makes activities meaningful for, and how they are experienced by, people with different types of dementia in their own homes. This study used focussed ethnographic methods to explore the motivations and meanings of everyday activity engagement within the homes of 10 people with memory-led Alzheimer’s disease and 10 people with posterior cortical atrophy. While participants’ interactions with their everyday environments were challenged by their diagnoses, they were all finding ways to continue meaning-making via various activities. The main findings are encapsulated in three themes: (1) The fun and the function of activities; (2) Reciprocities of care, and (3) The constitution and continuity of (a changing) self. Ongoing engagement with both fun and functional activities offered participants living with different dementias opportunities to connect with others, to offer care and support (as well as receive it), and to maintain a sense of self and identity. Implications are discussed regarding the development and delivery of tailored interventions and support to enable continued engagement in meaningful activities for people with different types of dementia living in the community.

## Introduction

### Dementia: Impact and Public Health Priorities

Dementia is an umbrella term used to describe any syndrome characterised by a progressive decline in cognitive function. This can include memory loss, problems with thinking, and changes in behaviour and leads to increased dependence in a range of everyday tasks over time (Cummings & Benson, 1983; Qiu et al., 2009; Rossor, 1994). The most common cause of dementia is Alzheimer’s disease, but it can be caused by a range of lesser-known diseases, many of which disproportionately affect people of a younger age (i.e. before the age of 65) (Rossor et al., 2010).

Dementia has significant economic and social costs to individuals, their families, and wider society (Wittenberg et al., 2019). Those with rarer types of dementia are likely to experience unique and complex additional challenges owing to the young age of onset and atypical symptoms associated with them. Posterior cortical atrophy (PCA) is one such rarer form of dementia. It is normally caused by the same underlying pathology as typical memory-led Alzheimer’s disease (tAD), but because the resulting damage disproportionately affects the back of the brain, PCA is primarily characterised by problems processing visual and spatial information, leading to dominant difficulties with seeing what and where things are, posing distinct challenges with a wide range of everyday tasks and activities (Crutch et al., 2017; Graff-Radford et al., 2021; Harding et al., 2018; Yong et al., 2023).

Despite recent encouraging developments with disease-modifying treatments for dementia (Edwards & Corkill, 2023), while we are without a cure for dementia-causing diseases, the public health priorities for people living with dementia (PLWD) include maintaining a good quality of life (QoL) and ageing in the right place, which for many PLWD remains their own home (Brittain et al., 2010; Chung et al., 2017; Von Kutzleben et al., 2012). One way in which QoL can be supported for PLWD is via continued engagement in meaningful activities, something which is reflected in policy recommendations and healthcare guidelines (e.g. National Institute for Clinical Excellence – NICE, 2018; World Health Organization – WHO, 2019).

### Theoretical Framework: Conceptualising Meaningful Activities

The individual variation in what can constitute meaningful activity (e.g. Hammell, 2004; Marshall & Hutchinson, 2001; Menne et al., 2012) is captured in a definition used by Travers et al. (2015), which states that “activities can be meaningful if an activity is somehow significant to, or valued by, the person and provides enjoyment, a sense of purpose, belonging or achievement” (p. 168). This definition was endorsed by PLWD and carers in Travers et al.’s (2015) study and is the one we use here. This definition is intentionally broader than that within the conventional and previously dominant discourse of active ageing, which prioritised activities associated with ‘doing’, and which are often associated with physical and/or cognitive effort (Adams et al., 2011). In including activities which can provide belonging and enjoyment alongside those which can provide a sense of achievement, Travers et al.’s (2015) definition inclusively acknowledges the potential value of activities which may be more passive too. This definition is also more compatible with the World Health Organization’s (WHO) more recent definition of healthy ageing (its strategic focus from 2015 to 2030) which, while still placing significant importance on functional ability, does also attest to the essential value of *being*, as well as doing, in outlining that “Healthy ageing is about creating the opportunities that enable people to be and do what they value throughout their lives” (WHO, 2020, p. 9).

To further foster this non-normative approach to activity engagement, a theoretical lens of everyday life was also applied to frame the study and analysis. At the core of the sociological approach to studying everyday life is a desire to gain an in-depth and contextualised understanding of people’s day-to-day experiences, and particularly relevant to the context of disability or illness, a need to critique and/or dismantle any pre-existing judgements and societal assumptions (Harvey, 2018; Scott, 2009). The theorising of everyday life allows for a more nuanced understanding of experiences, in which the meanings of the trivial, mundane, and unremarkable micro-moments can be elevated, which help with resisting the tendency to attend only to discretely divisible and traditionally defined tasks and activities (Hammell, 2004; Harvey, 2018; Hasselkus, 2006; Scott, 2009). It is through the disruption of the habitual, taken-for-granted, and familiar everydayness that profound personal impacts reveal themselves and can be understood (Scott, 2009). Attending to this complexity and variation can also prevent an illness experience being homogenised, which could marginalise those affected, something particularly worth consideration when studying those with underrepresented and lesser-known diagnoses as we are here (Harvey, 2018; Hasselkus, 2006; Scott, 2009).

### The Impact of Meaningful Activities: The Evidence Base and Knowledge Gaps

Maintaining engagement in such activities can promote well-being and meet fundamental psychological needs for PLWD including the maintenance of meaningful relationships and connectedness, fostering a sense of control, satisfying needs for creativity, providing opportunities to work towards goals, and offering continuity in one’s sense of self and identity (Allison et al., 2022; Han et al., 2016; Nyman & Szymczynska, 2016). Engagement in activities has been described as an essential component of a ‘good life’ for PLWD (Edvardsson et al., 2014) and was a key concept in Dröes and colleagues’ (2017) operationalisation of the ‘social health’ domain of the updated WHO definition of health for PLWD. Activity engagement can also help to reduce behavioural symptoms including apathy, agitation, and irritability and psychological symptoms such as depression and anxiety (e.g. Allan & Killick, 2000; Chung, 2004; Davies-Quarrell et al., 2010; Hewitt et al., 2013; Hydén, Lindemann & Brockmeier, 2014; Travers et al., 2015).

Activity which is individually tailored has been shown to be particularly effective (Sauer et al., 2016; Tierney & Beattie, 2020; Travers et al., 2015), and this is in line with the suggestion that what makes meaningful activities so important is the opportunity for personal meaning-making, value-seeking, and the expression of personal preference and choice that they provide (Chung et al., 2017; Strick et al., 2021). However, these idiosyncrasies can mean the evidence base is complicated, with a recent review concluding that the certainty of evidence is low and variable across outcomes including reducing challenging behaviour, and improving the QoL, affect and engagement of PLWD (Möhler et al., 2020). Additionally, most research into the benefits of meaningful activities has been focused on interventions delivered by others (meaning we don’t understand self-motivated activities well, despite the fact that most activities are self-motivated), in groups (making tailoring to the individual) and often most easily studied within more controlled residential care settings (meaning that generalisability to a community setting is unclear) (e.g. Fernández-Mayoralas et al., 2015; Kielsgaard et al., 2021; Tierney et al., 2023). Furthermore, this research has primarily focused on people over the age of 65 living with typical, memory-led forms of dementia such as Alzheimer’s disease (meaning we have little understanding of how meaningful activity engagement is impacted for people affected by young onset and rarer forms of dementia) (e.g. Allison et al., 2022; Fernández-Mayoralas et al., 2015; Tierney et al., 2023). In addition, the nature of activities which have mostly been studied are those which subscribe to the dominant aspects of discourses of active or healthy ageing in being effortful and centred on achieving, functional ability and ‘doing’ (meaning we may be missing more passive, deactivating, or restful activities which are more about being, and which may also be immensely subjectively meaningful for PLWD, particularly if their cognitive and physical capabilities are challenged) (e.g. Kielsgaard et al., 2021; Tierney et al., 2023).

In this study, we aimed to understand the subjective motivations and meanings of self-chosen everyday activities for people with either PCA or tAD, within their home environments, using focused ethnographic methods.

## Materials and Methods

### Epistemology

This study takes an interpretivist stance to data collection and analysis and a constructivist approach to any knowledge generation via this study, given the interest in the subjectively variable meanings associated with self-chosen activities and the importance of accounting for multiple (and potentially conflicting) perspectives within the data collection (e.g. PLWD and their family members) and analysis (e.g. a multi-disciplinary research team).

### Study Design

This study used a qualitative embedded multiple case study design (Baxter & Jack, 2008; Yin, 2017) drawing on focussed ethnographic methods. Focussed ethnography is a pragmatic and time-efficient variation of classic ethnography and is increasingly used in healthcare settings where extended immersion in the field of interest is not possible (e.g. because of resources or participant burden) (Knoblauch, 2005; Simonds et al., 2012; Wall, 2015). Although collected over a shorter period, the data collected in focused ethnography are often enhanced in terms of intensity and volume with the help of technological aids (e.g. video recordings) (Knoblauch & Schnettler, 2012; Pink & Morgan, 2013; Simonds et al., 2012; Wall, 2015). Further details of the methodological approach and justification have been documented elsewhere (Harding et al., 2021).

### Sample and Setting

Participants were recruited via the Cognitive Disorders Clinic at the National Hospital for Neurology and Neurosurgery, University College London Hospitals NHS Foundation Trust, a specialist hospital clinic, and the UCL Queen Square Institute of Neurology’s Dementia Research Centre, an affiliated research centre which both see patients/participants affected by a range of typical and rarer forms of dementia. Participants had to have capacity to consent to the study, a diagnosis of PCA (Crutch et al., 2017) or tAD (Dubois et al., 2014), and an accompanying carer. During or following their clinic or research visits, eligible participants were provided with information about the study and the opportunity to ask any questions before deciding whether or not they wished to proceed. Visits were conducted in participants’ homes across the United Kingdom by EH and were approximately 9 hours long (covering the morning and afternoon).

### Ethical Considerations

Ethical approval for the study was granted by the National Research Ethics Service Committee – London Queen Square (approval number: 06/Q0512/81). Extra care was taken to outline and discuss the specific ethical considerations owing to the methodologically innovative nature of the study (e.g. extended home visits and unstructured video-recording). All participants provided written informed consent.

### Quality Assurance

Emerging consensus asserts the value of concepts such as authenticity, trustworthiness, sensitivity, and utility (e.g. over standard checklists), in establishing the quality and rigour of qualitative research. In this study, we considered verification strategies outlined by Morse et al. (2002) throughout the whole research process. These included methodological coherence – ensuring congruence between the research question and methods – which was addressed in the selection of an exploratory method and inductive analytic process, given the little knowledge about the topic amongst the population studied here. Appropriate sampling was another criterion considered, and this included those with a lesser-known form of dementia and participants who were living in the community as opposed to residential care, due to the little knowledge about self-chosen meaningful activities in those with rarer dementias within their own home environments, and novel opportunities for intervention here. Morse et al. (2002) also suggested iterative, cyclical data collection and analysis procedures which move between the micro and macro levels, something we bore in mind in constantly moving between the individual ‘caseness’ and group-level patterns emerging from the data during analysis, in looking both within diagnostic groups and across the sample as a whole for commonalities, and in the creation of analytic memos throughout the data collection and analysis phases of the research.

### Reflexivity

A reflexivity journal was kept by EH throughout the study to document researcher impressions, assumptions, expectations, and biases (Jootun et al., 2009). Key considerations and reflections included assumptions about the dominance of the diagnosis and difficulties in everyday life given EH’s position as a researcher within a primarily biomedical research department. Given the research department’s biomedical focus, this study was a novel undertaking for the participants, many of whom are experienced with undergoing neuropsychological testing and experimental research studies at the host centre. Many participants had questions about our specific interests and what they ‘should be doing’ during the home visit or what would be ‘helpful’ for the researcher to observe. With a social science background and expertise in qualitative research methods, the lead researcher EH was able to address these questions within the process of the consenting conversation, to ensure participants had a good grasp of the ‘everydayness’ we were hoping to observe and why this was important and to check their level of comfort with this novel and unstructured approach. Despite this being clarified, there were inevitable limitations to how ‘typical’ a day participants could have while being observed by an unfamiliar researcher. For example, some participants seemed keen to highlight areas of difficulty in order to ask for advice or suggestions from the researcher. Others were keen to showcase strategies they had developed beyond those which were relevant for the particular activities they were undertaking on the day, in case these could be shared with others. One participant announced he had ‘saved up his weekly chores’ for the research visit day to ensure the researcher could gain insights into as many challenges and strategies as possible while there. These instances were important to note but did not discredit the data collection or study aims as our research questions were not to gain an accurate snapshot picture of exactly what people were doing day to day and how effectively but rather to understand the motivations underpinning and meanings made of their everyday activity engagement. The atypicalities mentioned above all provided opportunities for the researcher to probe further via informal interviews to elicit greater understanding of participants’ perceptions of and wants for the home visit and what this could illuminate about their relationship to activities more broadly. For example, the gentleman who consolidated his weekly chores into one day did so because of his desire to participate as helpfully as possible in the study so that as much as possible of his experiences could be shared with peers in a similar situation. Further conversation about this highlighted ‘being helpful and of service to others’ as key values he sought to embody within his day-to-day activity engagement. The unstructured nature of the home visit protocol and the lead researcher’s expertise in qualitative methods of enquiry permitted this further exploration in a flexible and iterative way which contributed to data richness and quality.

Data viewing sessions with different combinations of the research team played a critical role in enriching the analysis, due to the different specialisms within the team. For example, as research neuropsychologists, SC and KY noted with particular interest the distinct impairments demonstrated across the two groups, as well as the potential mechanisms underlying the effectiveness of any supportive strategies. As psychologists with more clinical leanings (e.g. health psychology, counselling, and social work), PC, JS, EH, and MPS. rounded out this focus on impairments and adaptations with their considerations of the meanings made of any observed challenges and the individual and relational processes which underpinned any efforts to compensate for these. Coming from a social gerontological perspective, MPS offered a theoretical lens on how the motivations towards and meanings made of given activities could be situated within broader dementia discourses such as that of living well, citizenship, and the social model of disability.

Further explorations of the reflexive process including the emotional impact on the researcher and challenges in maintaining the researcher role can be found in Harding et al. (2021).

### Data Collection

In line with previous focused ethnographic and home-based observational studies (Briggs et al., 2003; Higginbottom et al., 2013; Knoblauch, 2005; Knoblauch & Schnettler, 2012; Simonds et al., 2012), ethnographic data of the following types were collected:

• Observational field notes – brief notes taken by hand throughout the visit, written up in full within 24 hours.
• Visual ethnographic mapping – sketches to capture the layout of the home and salient participant use of the space (Causey, 2021).
• Video-recordings – participants’ activities and commentary were captured by 360-degree cameras (4K; 360FLY) and wearable clip-on cameras (VEHO HD; MUVI).

Methods of data collection which were sensitive to nonverbal expression and communication (e.g. embodied movement, facial expressions, and gesture) were considered of particular importance given participants’ potential difficulties with recalling or communicating their experiences in words, due to their diagnoses.

In addition to this, given our interpretivist epistemological stance and the multiple actors involved in the observations (PLWD, carers, and researchers), care was taken to note how and by whom activities were initiated (e.g. carer suggestion, PLWD action, and environmental cueing) to ensure that the nuanced and relational nature of motivation and meaning-making via activity engagement could be captured.

The home visits were unstructured to ensure that a non-normative view of activities was maintained, without any constraints imposed as to what would ‘count’ as an activity, in order to ensure we were not inadvertently prioritising cognitively or physically effortful activities.

Other data were collected as part of the larger study within which this sub-study was nested (e.g. self-report questionnaires and physiological measures) which will be reported separately.

### Data Analysis

Data analysis was an iterative process of organisation, classification, constant comparison, interpretation, and triangulation. As recommended by Briggs and colleagues (2003), joint data viewing sessions with researchers EH, MPS, KY, and SC were conducted, which provided opportunities for any assumptions, impressions, and interpretations to be jointly considered and for possible alternatives to be generated.

Data analysis was supported by Atlas.ti (version 7). Data were uploaded and indexed before a process of re-immersion and re-familiarisation. Codes were created, assigned, refined, and grouped accordingly, and recurring patterns and themes were noted. Memos about emerging analytic ideas were created throughout (Briggs et al., 2003; Roper & Shapira, 2000; Yin, 2017).

**Figure 1.**
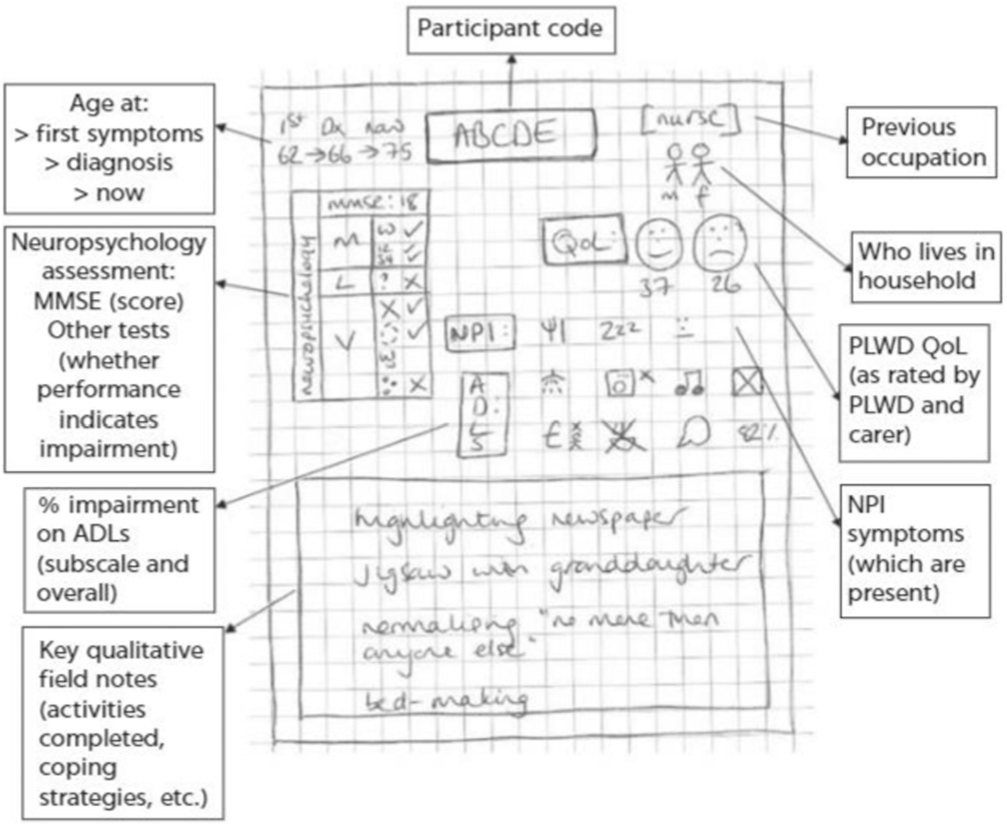
Non-identifiable example of index card.

Following the abstraction of the data through coding, efforts were made to find a way to visually represent the individual ‘caseness’ of each home visit (Yin, 2017). This eventually took the form of a series of index cards containing key observations and data segments relating to each participant (see Figure 1). These were viewed collectively in different combinations, for example, grouped according to diagnosis and the level of functional impairment. This allowed each individual case to be represented throughout the process of broader analysis across cases.

Once the thematic analysis was completed and key patterns identified, each individual case was written up using Geertz’s (2008) approach of ‘thick description’. This is a process of both describing and interpreting behaviour within a particular context. This acted as a final verification exercise in confirming the comprehensiveness of the themes.

## Findings

### Sample Characteristics

Ten people with PCA (6 males; 4 females) and 10 people with tAD (5 males; 5 females) took part in the study. Fourteen participants lived with their spouse only; two with their spouse and daughters; two with their spouse, daughter, son-in-law, and grandchildren; one participant was widowed and lived with his daughter and granddaughter; and one participant lived with his spouse and a lodger. Participants’ homes were in a variety of rural and urban locations across the United Kingdom and were a mixture of houses and apartments. Summary demographic information is available in Table 1.

**Table 1.** Summary Demographic Information.

Variable	PCA	tAD
Number of participants	10	10
Gender (male/female)	6/4	5/5
Age (years – mean ± SD)	67.6 ± 8.28	76.7 ± 6.13
Years since subjective onset (years – mean ± SD)	7.1 ± 3.14	6.5 ± 4.36
Years since diagnosis (years – mean ± SD)	3.3 ± 1.95	3.1 ± 2.26
Years between subjective onset and diagnosis (years – mean ± SD)	3.8 ± 2.68	3.4 ± 3.61
MMSE (mean ± SD)	17 ± 5.55	20.1 ± 5.45

### Qualitative Findings

Findings from the focused ethnographic observations revealed a range of activities that were meaningful to the person living with dementia, which have been organised into 3 themes: (1) The fun and the function; (2) Reciprocities of care; and (3) The constitution and continuity of a changing self. Each theme is described with illustrative examples and data extracts and is supplemented with biographical information about participants to situate the data in the contexts of participants’ life stories (including additional information about participant’s age, gender, and diagnosis in parentheses).

#### Theme 1: The Fun and the Function

This theme captures the variation in types of activities that participants engaged in, as well as the varying possible outcomes of activities and the meanings these held. Participants in both groups were motivated to continue to engage in a variety of activities that were both fun and functional, even if this had to be in necessarily modified ways. As well as engaging with a range of both fun and functional activities, the observations illuminated the importance participants appeared to place on the *process* of engaging with activities (i.e. the ‘fun’ of doing them), as opposed to only the outcome (i.e. the ‘functional’ output of them).

All participants were retired, and as such there were large portions of the day, outside of the completion of essential ADLs such as washing and dressing, in which people found ways to engage with activities they found pleasure, enjoyment, interest, satisfaction, fulfilment, and/or other forms of meaning in. These included gardening, listening to and playing music, watching television, reading, and doing puzzles. The more ‘functional’ activities completed by participants in both groups included laundry, washing dishes, and hoovering. Participants had varying individual preferences for and motivation towards more fun or more functional activities, which often related to previous roles or identities, or were described as components of their longstanding character or personality. For example, Alan, a retired sound-system installer, expressed (partly verbally and in demonstrating with his hands) how satisfying he found the hands-on work of untangling and reconnecting the heaps of wires his customers were often overwhelmed by. Alan supported this further when clearing dead leaves from the garden by hand and expressing with emphasis “I want to do something,” while gesturing with his hands an action that implied getting ‘stuck in’. Of his preference for more ‘functional’ activities, Alan’s^1^ wife said:

Occasionally he puts his super expensive headphones on and sits in that swivel chair there and listens to a CD there but … he thinks … I think he thinks it’s wrong to sit down and do nothing … he’s sort of always thought he should be doing things and it’s wasting time to sit and relax. So he’ll be up doing what he’s doing now [picking up leaves] rather than sit and listen to music. (Wife of Alan, 64, male, PCA)

The fun and the functional were not mutually exclusive – activities could be functional and still provide a sense of pleasure and enjoyment and/or fulfilment and meaning. For example, dressing and grooming appeared to be an important functional activity, which held significant personal meaning for Eleanor (74, female, PCA), and was not just a task to tick off. Eleanor was dressed in various shades of blue on the day of the home visit, and there was a selection of numerous other blue cardigans and jumpers laid out on the bed, highlighting the thorough selection process Eleanor had been through that morning before deciding on her outfit. This evident effort was arguably an indication of the importance she placed on this activity along with the value she placed on her independence in it. This was further supported by Eleanor’s apparent resistance to her partner’s input with helping to get items from the wardrobe and her offers to place them back once they’d been decided against. Situating this within the context of Eleanor’s identity and relationship, Eleanor had explained that her independence had always been extremely important to her and was now challenged in almost all everyday activities because of her dementia-related visual problems:

I suppose the biggest problem for me these days is my eyes – because once you can’t see ... it shuts off everything else for you.

///

I miss that freedom, when you’ve been independent ... as most people are in a lot of their lives, you miss it when it goes… (Eleanor, 74, female, PCA, MMSE: 20)

Eleanor’s present resistance to her partner’s attempts at strategies to support her dressing could also be situated in relation to Eleanor’s character (“I’ve always been challenging ... and long may I challenge!”) as well as the longstanding nature of their relationship (“don’t worry, we’ve always been this abrasive!”), demonstrating how the construction of meaning of everyday activities could be underpinned by longstanding individual and relational qualities and ways of being.

Even though dressing wasn’t an efficient activity for Eleanor, it seemed to provide sufficient meaning that it was persevered with despite the challenges faced. More broadly, common priority outcomes for activity completion such as accuracy, efficiency, or speed appeared to be less significant for both groups than the sense of purpose, meaning, connection, or enjoyment that the process of engaging with such activities could provide.

Another example of this kind of alternative outcome being prioritised was observed with Richard’s (77, male, tAD) difficulties with loading the dishwasher. Part way through the process, Richard began moving items straight from the sink onto the draining board, bypassing the dishwasher altogether. This meant that Richard’s wife then had to later return to the task to correct Richard’s attempt, by double checking that everything that needed to be washed had indeed made it into the dishwasher. In this and many other examples observed during other home visits, it would have arguably been much more efficient for the family member (Richard’s wife in this example) to have completed the task herself in the first instance, so the fact that this is not what happened attests to there being something else to be gained from Richard’s engagement in this activity, an alternative priority outcome such as his engagement and involvement in daily routines. This suggestion that there was some meaning for Richard in completing tasks like this was supported by his repeated and proactive approaches to his wife throughout the home visit to ask what he could do to help and in his asking for demonstrations of the various household chores he was assigned. This also points to the relational aspect of activity engagement for PLWD (as will be further expanded on in Theme 2), as while Richard himself may have experienced this activity as being completed efficiently, the experience (and associated priority outcomes) was different for his wife.

Further testament to the meaningfulness of participants’ self-chosen activities was the perseverance they showed in continuing to engage despite the challenges faced, albeit often in modified ways. A poignant example came from Martin (73, male, PCA), a retired university Head of Education who had always enjoyed birdwatching and contributing to community efforts to support local bird-life, before his diagnosis. Martin’s wife explained that he now had difficulty perceiving larger or stationary birds, owing to his PCA symptoms. Martin described and demonstrated the multiple modified ways in which he continued to engage with this activity of interest throughout the home visit, in enjoying watching and feeding smaller birds in the garden from his kitchen window, by looking through books with detailed illustrations and descriptions of birds, and by taking time to look at and share with me greeting cards illustrated with different birds displayed around his home.

Many participants modified their mode of engagement by engaging socially in the topics or activities they were no longer able to physically engage with, for example, with storytelling about the past or verbally communicating planned activities. Betty (88, female, tAD) had previously been a model, and many of the activities she habitually engaged with or indicated interest in related to aesthetics, for example, she spent a long period tending to her cuticles (even though her nails were already beautifully painted), repeatedly described her garden makeover plans, became very animated in conversation about the valuation of the artworks on the wall, and repeatedly expressed her desire to help clear away after lunch. This highlighted how longstanding meanings associated with activities across the life course could still be expressed, even when functional abilities such as mobility and memory difficulties could inhibit the actualisation of these intentions and execution of these activities.

Occasionally, priorities for activity engagement were conflicting or incompatible, as illustrated by Maurice (79, male, PCA) and his wife’s differential interpretations of him spending his afternoon dozing, which also impacted how these activities were encouraged or motivated differently for carers than PLWD. For Maurice’s wife, his dozing was considered almost a luxury, which was also helpful as it permitted her time to get on with errands, but Maurice was visibly saddened when he explained, for him, having a nap as being a waste of his time, and how much he missed being as busy as he had been in his fast-paced and demanding career. Maurice had been a print room floor manager often working throughout the night and described how energising he had found that work. Outside of work, many of his longstanding hobbies also connected to the value of productivity, for example, model engineering and metalwork using a lathe, illustrating again how the meanings and motivations towards activities exhibited or expressed during the home visits were often biographically situated. This was one of many examples of the relational nature of how activity engagement was experienced – explored more thoroughly in Theme 2.

#### Theme 2: Reciprocities of Care

This theme captures how activities were a mechanism by which care could be both delivered and received and importantly that this didn’t happen only in one direction. Activity engagement was often an inherently relational process – carers in both groups played an essential role in supporting their family member with dementia to continue to engage with activities, from activity selection (e.g. orienting to task) through to execution (e.g. verbal guidance or physical assistance). While this support was mostly characterised by reciprocity and aligned priorities (e.g. with carers encouraging engagement in activities that maintained the PLWD’s sense of identity, as discussed in Theme 3), the relational nature of support with activity engagement also highlighted some differences in motivations and meanings made of activities, within dyads. For example, occasionally motivation towards an activity would appear solely driven by a carer who would seem to be encouraging the PLWD’s engagement in an activity they felt would highlight any difficulties and therefore their support needs most representatively, or which would exercise particular skills they were struggling with in order to improve these. For example, Rhian was encouraged to unstack the dishwasher and hang laundry which highlighted her difficulties with spatial awareness, and Lilian was encouraged to try to name family members in photographs shown on an iPad, which proved difficult due to her perceptual problems being exacerbated by reflective glare.

Engagement in activities was also observed as a key way that PLWD delivered care or made a contribution to others. Poignant examples of this came from Lilian describing the reciprocal relationship she had with her paid carer and how meaningful she was finding their interactions and activities they undertook together:

She’s absolutely lovely and we’ve become great friends, and because she’s different, she’s very interesting … She’s lovely. I take her … to places she’s never seen, ‘cause I’ve lived here I know the area. (Lilian, 70, female, PCA)

The longstanding importance of activities which enabled social connection with others for Lilian was highlighted when she expressed her sadness at no longer being able to independently travel into town to see people. This particularly valued aspect of activities was something Lilian demonstrated continued keenness to engage with throughout the day, when enquiring in great detail about people who telephoned, in comparison to when she was left alone to watch television, during which she appeared much less engaged.

Testament to the variable meanings made of different activities, television-watching for Mandy (72, female, tAD), appeared to be much more engaging and socially connective. We spent several hours watching the Winter Olympics with Mandy’s husband, and Mandy regularly used the action on the television to prompt interactions with us (e.g. “could you do that?”). This also highlighted the potential for what we may consider to be more passive activities to be meaningfully engaging for PLWD.

Another participant for whom the meaningfulness of activity had a social component was Lionel, who had spent many years prior to his diagnosis as a full-time carer for his granddaughter with complex health needs. Lionel continued to prioritise measuring out his granddaughter’s medication as one of his regular activities (overseen by his daughter), along with frequently checking in on her. Lionel’s prioritisation of activities which were supportive to others was also evident in his daughter’s comment:

All he wants to do is help ... do you know sometimes he comes up to me and salutes and says, “your humble servant is here what can I do for you?” ... he’s not interested in doing anything just for himself. (Lionel, 76, male, tAD)

A number of participants like Lionel had existing caring responsibilities, and as a result many of the activities they engaged in during the observation period were inherently acts of care towards others. Anita (88, female, tAD), a retired special educational needs teacher, had for a long time prior to her diagnosis been the primary carer for her husband of more than 60 years who was registered blind. Her husband summed up the recent shift in their now more reciprocal helping relationship when he explained “I provide the memory and she provides me with eyesight.” Anita continued to engage in many care-taking activities such as preparing food and drinks for her husband despite her difficulties (e.g. Anita demonstrated some confusion about a jar of coffee by turning it around in her hands several times with a puzzled expression). One of the self-chosen activities Anita was independently motivated towards during the home visit was playing the piano, which also seemed to offer a sense of meaningfulness from doing things for others, as Anita asked her husband which songs he would like to hear her play.

Another way this desire to help or to provide care manifested was in participants’ concerns about being a burden to their primary carers and adjusting their expectations or requests around activity engagement in response to this. For example, Maurice (79, male, PCA) expressed a desire to donate his large collection of computer magazines to others who may be able to use them now that he was unable to read them, but expressed frustration that he couldn’t orchestrate this himself, and a reluctance for this to become another task for his wife to organise on his behalf. Martin (73, male, PCA) expressed similar concerns about being a burden to others when he described withdrawing from his local dance class because it involved switching partners and he was concerned about letting others down by not being able to keep up with the steps and adjusted his activity engagement accordingly by dancing in the kitchen at home with his wife instead.

For several participants, an essential way in which they found the reciprocal nature of activities to be meaningful was in the sense it gave them of being part of a team. Mark (68, male, tAD), a retired paediatric neurologist, described how much he missed the team-based activities he had participated in throughout his life, from working behind the bar throughout medical school, to rugby-playing, to his later occupational team as a neurologist. He further captured the meaningfulness of team work when he said “if you work together you can do things”. Reflecting on his and his wife’s joint efforts (e.g. her reminding and him actioning) in negotiating his current activities, he added, “once you’re married that’s the team, isn’t it?”. Martin (73, male, PCA) described how even playing a small part in an activity – occasionally filling the communal neighbourhood birdfeeders – that he knew mattered to others, could foster positive feelings for him, which seemed to stem from a sense of connectedness to others around a subject of shared interest. Together, these examples also highlight the significance of relationships beyond the caring relationship for PLWD’s meaningful activity engagement.

A final way participants found to be contributory in their activity engagement was in their research participation itself. Wendy (75, female, tAD) summed up the inherently generative motivation for participation which many participants expressed when she said “I don’t mind what I do if I’m helping you and other people.” This current generativity of Wendy was consistent with the keen interest she had shown in her grandchildren throughout the day (e.g. enquiring after them and completing a jigsaw puzzle with a visiting granddaughter), as well as the care and concern she demonstrated towards her two Burmese cats (e.g. regularly checking their whereabouts and grooming them both with her husband in what they affectionately called the ‘cat salon’).

#### Theme 3: The Constitution and Continuity of a Changing Self

This theme captures the various ways in which the activities people did (both intentionally as well as in habitually, unconscious, and embodied ways), had previously done, wanted to do, or planned to do, and the objects and spaces within their homes that connected to those activities appeared to constitute elements of the PLWD’s sense of self and identity.

The activities themselves often connected to longstanding personal hobbies or interests, sometimes to participants’ professional backgrounds, or to the family or other social roles that were important to them. With independent engagement with these activities often challenged, the objects and spaces associated with these sorts of meaningful activities could be seen to act as a reference point for these aspects of people’s identities and offered an additional opportunity for their sense of self to be represented and acknowledged.

For example, former model and beauty retail assistant Lilian had long prioritised activities relating to aesthetics in both her professional and home life including grooming, fashion, and interior decorating. The connection these activities had to her enduring sense of self and identity seemed to be captured when during a tour of the home she commented:

I used to love all the antique shops and things … I just miss being the woman around the house, you know. Doing things my way. I do miss it … that’s what you do when you’re at home, don’t you? You do things, whereas I don’t do things anymore and I miss it … it’s nothing like being your own self is it? I used to like to have all my pictures up on the wall … I used to like it all … I still like it yes, I can’t do any of it. (Lilian, 70, female, PCA)

Lilian articulated the link between the things she was able to do and her sense of being her ‘own self’ and saw her ability to organise her physical environment and the objects within it in a way that reflected that self as an essential expression of her personhood. Like other participants, Lilian was continuing to persevere with the activities she could manage: for example, she spent an extended amount of time on her morning routine applying creams and checked the ingredients for dinner although she wasn’t involved in the cooking. This perseverance in continuing to engage with meaningful activities in modified ways despite the challenges was testament to participants’ overall drive to maintain their interests, activities, and the sense of identity these constituted.

The adoption of perseverance and other supportive strategies was contingent on a number of intra- and interpersonal factors such as personality, previous or existing relationship dynamics, and individual preferences, for example, independence. Explaining her preferences for independence in her activity completion, Eleanor commented:

I would like to go out for walks on my own, I would like to drive a car, I would like to have a life ... I miss that freedom, when you’ve been independent ... you miss it when it goes. (Eleanor, 74, female, PCA)

Eleanor detailed the fundamental importance of independence in the choice and execution of activity engagement for her sense of having ‘a life’, and how difficult the change in that independence had been to adjust to, when it had previously been so defining.

Identity maintenance was also relational in many cases, with participants’ carers or family members often enacting this on behalf of the PLWD, via activity engagement. One way this was observed during the home visits was with carers deferring to the PLWD’s expertise. For example, Wendy’s (75, female, tAD) husband consulted her – as the person who had done most of the household cooking for many decades – on the recipe for lunch and asked for her advice on the timings for cooking, so although Wendy was not able to initiate or manage the cooking as she had done previously, her husband had acknowledged her expertise from her years of experience of cooking and provided an opportunity for it to be recognised. Other ways carers acted to maintain the PLWD’s identity via engagement with meaningful activities included eliciting and prioritising the PLWD’s preferences and choices, and by minimising any diagnosis-related difficulties that were evident during activity completion (e.g. by normalising).

Another aspect of this final theme was the way changes in participants’ engagements with activities over time related to their identity and sense of self. A poignant example of how these changes in oneself could be constituted by the environment itself and the activities undertaken within it came from Sally’s explanation of her regular pastime of looking out of the window of their rural bungalow and articulated the meaning of this activity for her in the context of her condition and the associated changes:

I have places where I can come and sit ... I like the ones with the sun or sunset, sunrise and sunset are the really good ones. ‘Cause I just like looking at the edge [horizon]. It’s been, can’t remember how long we’ve been here but it’s so familiar, and it’s not gonna shift. I mean the [hills] aren’t gonna fall down. It’s something that is not going to disappear quickly ... It’s not permanence because nothing is permanent but it’s a … it’s like an anchor. (Sally, 64, female, PCA, MMSE: 19)

This example highlights the constituting capacity the physical environment can have in that the activity of watching the sun rising and setting offered some acknowledgment of the changes within Sally, but also the possibility for some continuity and comfort, during this period of inevitable change. This was also a poignant example of what may appear as a more passive activity, a way of being, perhaps not always appreciated within the conventional and dominant aspects of active and healthy ageing discourses, but one which provided significant personal meaning.

## Discussion

This study explored the importance of continuing engagement in both fun and functional activities for community-dwelling people with different types of dementia, and the valued opportunities these provide for maintaining one’s sense of self and identity and for fostering ongoing reciprocity, whereby PLWD can continue to offer care and support to others (as well as receiving it from them).

The modified modes of engagement that people developed in order to continue with activities are also reflected in other empirical literature and include talking about previous meaningful activities or plans for future activity participation, holding or sharing objects or photographs associated with meaningful activities, enquiring as to others’ feelings about a given activity of importance, and imagining or envisioning future activity engagement (e.g. Boyle, 2014; Emirbayer & Mische, 1998).

The perseverance people showed despite any inefficiencies or difficulties with activities is compatible with findings from other studies. For example, Phinney and Chesla (2003) reported that when PLWD begin to experience their usual skilled and habitual bodily actions as being disrupted and start to have difficulties in holding thoughts and words in mind, they adjust the attention and effort they apply, taking extra care in order to continue to do those things which previously felt effortless and automatic. This echoes Phinney et al.’s (2007) findings that PLWD were motivated to continue engaging in household chores even when these became increasingly difficult and Menne and colleagues’ (2012) unanticipated finding that PLWD were still motivated to engage in cognitively demanding activities such as reading and writing even though their condition is one of cognitive impairment.

The current study findings emphasise the importance of the process and experiences of activities, as opposed to just the outcomes, and the often relational nature of this process is also reflected in other studies. Merrick et al. (2016) described a participant who continued to help scaffold her husband’s involvement in food and drink preparation even though this often resulted in a mess in the kitchen that she would need to spend time tidying up. Edvardsson et al. (2014) also encouraged a similar focus on the process of activities rather than the outcomes for personal care-related activities and suggested a reconceptualisation of care-task endpoints to meaningful moments between individuals.

The importance of the carer role in the framing of activity engagement and outcomes has also been demonstrated by Majlesi and Ekström (2016) in their study of couples baking together. The authors suggested that PLWD responding to instructions from a carer is more positively and helpfully framed as a collaborative and contributory act rather than a sign of the PLWD’s incapacity and reliance. Similarly, they suggested that conceptualising PLWD’s reassurance-seeking as an indication of their motivation to avoid mistakes and an appropriate understanding of their potential to make them, rather than as an indication of their failings, offers a more enabling interpretation.

This perseverance and prioritisation of the process in the face of challenges illustrates that the meanings made when engaging with activities extend far beyond simple enjoyment, and this too resonates with other studies. Chung and colleagues (2017) concluded that activity selection is often not driven by any particular outcomes or successes but by the opportunities they offer the PLWD for some continuation in their sense of agency, by connecting them to long-held interests or roles. Aligning with this, Nyman and Szymczynska (2016) demonstrated how such activities went beyond pleasure-seeking to meet some fundamental needs of PLWD, including reflection over and connection with one’s life history, the fostering of intergenerational relationships, and the opportunity to feel a sense of being in control.

These needs were met by a range of activities in the current study, and while there were common tendencies towards activities which helped others or promoted continuation of one’s sense of self and identity, there were also a wide range of other motivations for activity engagement, including activity for occupation, familiarity, entertainment, education, comfort, and relaxation. This variation in motivation is endorsed by Øksnebjerg and colleagues’ (2018) work which looked at what PLWD wanted to gain from psychosocial interventions, and which included confidence, feeling good, feeling competent, having fun, feeling useful, and feeling equal to others.

The wide variation in the self-chosen activities observed and their meanings are consistent with other study findings that meaningful activities which can be tailored to individuals have the greatest impact on well-being and other positive outcomes (e.g. Lokon et al., 2019; Sauer et al., 2016).

This study builds on previous work by illustrating that the underpinning themes of what it is that constitutes meaningful activity did not differ across diagnostic groups even though symptoms and challenges with everyday activities may do (e.g. Harding et al., 2018; Shakespeare et al., 2015; Suárez-González et al., 2016). The findings of this study also support but build on those of Allison et al. (2022), by highlighting that continuity in the meaning of activities across the lifespan and the sense of identity they foster is also experienced by those experiencing dementia at a younger age and with different subtypes. These findings have also delineated and disentangled – and in doing so perhaps endorse, while helping us to move past – some of the outcome-oriented confusion in the existing meaningful activity literature, by demonstrating that outcomes for meaningful activities are dynamically renegotiated and reprioritised across time, activity type, and family systems. In taking a broad non-normative definition of activity and utilising unstructured observational methods in order to capture these, this study has also highlighted – counter to the traditionally dominant aspects of the active and healthy ageing discourses – the significant individual meanings that can be made via a range of self-chosen activities which may be passive, restful, and low in physical or cognitive demands. The study findings also extend previous work by highlighting the specific importance of meaningful activity engagement for those living with dementia at a younger age given the different identities which can be threatened, and which PLWD are motivated to maintain, while still of working age, when non-memory-led dementias like PCA (as studied here) are more likely to impact people. As well as illustrating the supportive aspect of the relational context in which activities are engaged with, in being sensitive to the multi-layered perspectives and priorities of PLWD, family members, and researchers observing, this study has also highlighted some potential complexities of the relational aspect of activity engagement within naturalistic settings, for example, how these different preferences, priorities, motivations, and meanings are negotiated and interpreted. Finally, this work emphasises a specific relational component of meaningful activity engagement which is little-studied – a legacy component – adding an important dementia-diversity angle and voice into the developing conversation about what it is that PLWD can and want to *give*, which can sometimes be lost amongst the efforts to understand and deliver what PLWD need or want to receive (Hollinda et al., 2023; Johnston & Narayanasamy, 2016; Nimmons et al., 2023).

### Limitations

Conducting the observations within the home environment may have contributed to some sampling bias in that dyads who were not managing well may have been less comfortable with being observed and may have declined participation. Potential participants who were further into their disease course and therefore more impaired and more likely to exhibit behaviours that challenge (e.g. agitation) may also have been selected against. Another way in which our sample may have been biased is in terms of socioeconomic status, with most participants having a high level of education, occupational status, and financial security. Most participants were also of white British ethnicity. All of these factors could affect the motivations and meanings underpinning activity engagement for these populations, and future work with more representative and hard-to-reach groups will be important. Characteristics of the visiting researcher (white British female in mid-thirties) may also have played a role in shaping the sample.

Additionally, in being conducted at a single time point, these findings provide a snapshot of participants’ experiences with activity engagement. Although participants often referred to their previous and anticipated activity engagement, future longitudinal work would be helpful to corroborate this. Finally, while efforts were made to ensure participants’ own constructions of meaning were captured (e.g. in attending to nonverbal and embodied expressions of motivation and meaning-making), there may still be an over-representation of researcher and carer perspectives on the motivations and meanings associated with PLWD’s activity engagement, particularly when these may have appeared somewhat ambiguous and required further interpretation. This is a widely acknowledged challenge for researchers seeking to access and report the lived experiences of PLWD, is of particular importance in the study of something as subjective as meaningful activity (Chung et al., 2017; Strick et al., 2021; Travers et al., 2015), and is something we have attempted to take small steps in addressing by utilising observational methods alongside more traditional interviews to maximise PLWD participation (e.g. Nygård, 2006).

### Implications

These findings have highlighted the feasibility and value of conducting focused ethnographic research with people living with different types of dementia in their everyday home environments. Observational work in naturalistic settings can offer unique insights, high in authenticity which capture the complexity and nuance of lived experiences in a way that is accessible for participants with a range of cognitive profiles. Given the distinctly different symptom profiles of those with rare and young onset dementias (e.g. Millenaar et al., 2016) and their relative underrepresentation in research, further studies exploring the needs and experiences of these groups will provide important contributions to the development of much-needed tailored support provision (e.g. McIntyre et al., 2019).

In the novel light they have shed on activity engagement for this understudied population in this understudied setting, the findings reported on here bring to the fore important questions in terms of the sorts of activities which are prioritised for PLWD and by whom (e.g. PLWD themselves, carers, researchers, and support staff), the different elements of process/outcomes of both fun and functional activities which are prioritised and for/by whom, and how discrepancies in these priorities can best be negotiated and managed within families, and hopefully provide useful steers and examples of the sorts of modified ways PLWD can be supported, encouraged, or facilitated to continue to engage with activities of meaning and importance within their everyday environments. Providing psychosocial interventions to support engagement with activities that promote one’s sense of self and identity and also those which offer the possibility of generativity (e.g. Doyle et al., 2015) may be especially helpful. Offering opportunities for a legacy component within interventions is one way these needs may be addressed – something which Johnston and Narayanasamy (2016) have suggested is an important but often-missed opportunity in the current delivery of psychosocial interventions. Finally, in adopting a broad definition of what constitutes activity, the study findings may also be helpful in informing interventions to support engagement with activities that may be more accessible and inclusive for PLWD, which may relieve carers feeling a sense of pressure to ensure PLWD are actively engaged in cognitively and/or physically demanding activities (Chung et al., 2008).

## Conclusions

The current study adds an ecologically valid perspective on the varied motivations, meanings, and priority outcomes of everyday activities for people affected by different types of dementia within their naturalistic home environments.

These findings may be helpful to PLWD, their families, healthcare professionals, and practitioners in supporting ongoing activity engagement in meaningful everyday activities and in turn fostering continuity in PLWD’s senses of identity and agency in making a contribution to and impact on the world around them.

## Data Availability

Due to the nature of this research, the data are not available due to their containing information that could compromise the privacy of research participants.

## Note

1. All names have been changed.
